# Hysteria

**Published:** 1882-04

**Authors:** Wm. Goodell


### HYSTERIA.

When called to treat a young girl with a hysterical attack, there are three things which you had better do: (1.) Institute at once firm pressure in the neighborhood of both ovaries. This is very apt to quiet the patient at once. (2.) Administer an emetic. I have found that a woman who is well under the action of an emetic has not the opportunity to do anything else than be thoroughly nauseated. Give a full dose of ipecac with one grain of tartar emetic. (3.) And this method of controlling the spasm will often act charmingly: take a good-sized lump of ice and press it right down on the nape of the neck. This produces quiet by its powerful impression upon the whole nervous system.—*Dr. Wm. Goodell, in Clinical News.*

